# Pharmacological Approaches for Management of Child and Adolescent Obesity

**DOI:** 10.4021/jocmr2010.05.288w

**Published:** 2010-06-15

**Authors:** Amar Kanekar, Manoj Sharma

**Affiliations:** a Department of Health Studies, 200 Prospect Street, Denike 14 B, East Stroudsburg University of Pennsylvania, PA, USA; b Health Promotion and Education, University of Cincinnati, P.O. Box 210068 , Cincinnati, OH, 45221-0068, USA

## Abstract

Overweight and obesity among children and adolescents continue to be a global public health epidemic. Current national data on childhood and adolescent obesity show alarming statistics of overweight and obese children and adolescents. This epidemic runs across various continents and affects various ethnic populations. The current weight management practices involve dietary modification, behavioral change therapies involving exercise, pharmacological therapy and surgical intervention. The purpose of this current review is to focus on pharmacological therapy i.e. role of sibutramine and orlistat in childhood and adolescent weight management. An open search of PUBMED database was made with search 'key words' such as 'orlistat' or 'sibutramine' or "pharmacological approaches" and "child obesity" and "adolescent obesity". This yielded a total of 20 articles. All of these articles have been summarized in the current review. Sibutramine functions by promoting satiety and increases energy expenditure by inhibiting reuptake of noradrenaline and serotonin. Most of the studies, reviews and trials conducted using sibutramine among adolescents and children show limited short-term efficacy. The long-term effects of sibutramine use are not yet studied due to the severity of its side-effects profile. Orlistat was approved by Food and Drug Administration for adolescent weight reduction. Despite its approval, it has a limited role in adolescent and pediatric obesity reduction due to the purported malabsorption of fat soluble vitamins and its side-effect profile. Obesity and adolescent weight management in present times mainly deals with dietary modification with superadded behavioral therapies promoting exercise. There is insufficient evidence as of now if these alone would cause adequate weight reduction and weight maintenance. Pharmacotherapy i.e use of drugs like sibutramine and orlistat has a limited role in the current fight against childhood and adolescent obesity. Extreme side-effects, close monitoring and lack of long-term studies involving these drugs, suggest questionable efficacy in current times. Future research studies involving pharmacological agents need to not only have immense scientific rigor during preliminary analyses but should also translate their efficacy in practical and clinical settings.

**Keywords:**

Obesity; Overweight; Adolescents; Pharmacological; sibutramine; Orlistat

## Introduction

Overweight and obesity continue to cause public health crisis in the United States. The recent National Health and Nutrition Examination Survey (NHANES) data reflects it to be quite a significant burden with 16.3% of all children between the ages of 2 to 12 years being found obese and 31.9% found to be overweight [1]. The rates in adolescents showed a worse picture with 17.6% being obese and 34.1% overweight. Further stratification of this data as per gender, adolescent males had the highest prevalence of overweight and obesity among all age groups, with 18.2% male and 16.2% female being obese and 33.3% female and 34.9% male being overweight. Overweight and obesity issues pervades across different ethnic groups such that non-Hispanic blacks (22.9%) adolescents have a higher reported prevalence when compared to non-Hispanic white adolescents (16%) [1]. The obesity and overweight epidemic though on rise in the United States doesn't exclude countries worldwide. In 2003, 23.5% Eastern Mediterranean, 25.5% European and 10.6% of South East Asian children and adolescents were reported either overweight or obese [2].

In the United States, obesity estimates among adolescents are often based on body mass index (BMI) percentile scores, which the International Obesity Task Force accepts as a valid method for measuring body composition. A recent meta-analysis of school-based obesity interventions however concluded that BMI may not be a modifiable outcome [3]. Adolescent body fat composition can be measured by triceps and sub scapular skin fold thickness. Investigators reported that child and adolescent body fatness has increased 0.9% per decade since the 1950s and in 2003, the average percentage of body fat for child and adolescent boys and girls were 16.2% and 22.2% respectively [4].

There are four modalities for current management of adolescent overweight and obesity: dietary management, increasing physical activity, pharmacological therapy and bariatric surgery [5-8]. The purpose of the current article is to review current pharmacotherapies for management of child and adolescent obesity.

## Role of Pharmacotherapy

In the United States, the Food and Drug Administration has approved two drugs for pharmacological therapy [7], these are orlistat and sibutramine. Orlistat blocks absorption of fat in the intestines by decreasing action of lipase and as a result creates negative energy balance. The main adverse effects associated with its use are malabsorption of fat soluble vitamins and oily stools. Sibutramine promotes satiety and increases energy expenditure by inhibiting reuptake of noradrenaline and serotonin. Sibutramine also causes anorexia. Side-effects of sibutramine include palpitations, high blood pressure and headaches.

## Methodology

The current review addresses role of orlistat and sibutramine as pharmacological interventions for management of child and adolescent obesity. The search methodology used PUBMED database with a Boolean search strategy including years 2007-2010. The key words of "orlistat", or "sibutramine" or "pharmacological approaches" and "child obesity" and "adolescent obesity" were used. This yielded a total of 20 articles, Table 1. Results of the current pharmacological trials, interventions, concepts and adverse effects of these two important drugs are summarized in the results section.

**Table 1:** Pharmacological Approaches and Other Related Studies for the Management of Child and Adolescent Obesity-2007-2010

Authors/Year	Type of study	Methods	Results	Conclusion
1) Rogovik study/2010 [27]	Review	Review of current literature related to role of pharmacotherapy and weight loss supplements in childhood obesity management	Orlistat is the only FDA approved drug among the group of weight loss medications for the treatment of overweight and obese adolescents.	Researchers conclude that no single approach will successfully treat obesity, and lifestyle modification presently remains the main pillar of any intervention aiming at decreasing bodyweight.
2) Whitlock study/2010 [26]	Systematic review	Researchers reviewed database of abstracts	Two medications showed significant weight loss	Short-term benefits of comprehensive medium- to high-intensity behavioral interventions in obese children and adolescents.
3) Coutinho study /2009 [10]	Review	Review of several studies using combination therapy of orlistat and sibutramine	It will be plausible to combine both drugs for beneficial effects.	Orlistat has proven its efficacy in the XENDOS study. Its role in the SCOUT study is yet awaited.
4) Czernichow study/2009 [17]	Meta-analysis of Randomized-controlled trials.	Total of eight trials with sample size of 1391 individuals was included in the present analysis	The mean decrease in weight between the intervention and the control group was 5.25 kg (95% CI 3.03-7.48) after a minimum follow-up of 6 months.	There was little evidence that treatment was associated with risk on cardiovascular risk factors.
5) Kanekar study/2009 [3]	Meta-analysis	Electronic databases were searched for school-based childhood obesity interventions and a total of five studies were analyzed.	The results of the pooled estimate of reviewed studies were not significant for the outcome measure body mass index at p < 0.05 level.	Current school-based childhood obesity interventions do not seem to modify body mass index
6) Tziomalos study/2009 [11]	Review	The most important articles on sibutramine up to January 2009 were located by a PubMed and Medline search	Sibutramine reduces food intake and body weight more than placebo	Sibutramine, in conjunction with lifestyle measures, is a useful drug for reducing body weight
7) Godoy-Matos study/2009 [6]	Review	Review of non-pharmacological, pharmacological and surgical treatment for obesity.	Pharmacological and surgical management is effective in weight reduction.	Sibutramine and orlistat may be good pharmacological options when lifestyle modifications alone do not work.
8) Oude Luttikhuis study/2009 [19]	Review	Review of randomized controlled trials of lifestyle, drug and surgical interventions was done from 1985-2008	Reduction in overweight at 6 and 12 months follow up was seen with or without the addition of orlistat or sibutramine	Combined behavioral lifestyle interventions can produce a significant and clinically meaningful reduction in overweight
9) Rogovik study/2009 [21]	Review	Review of weight-loss supplements and role of pharmacotherapy	Pharmacotherapy should be considered for children older than 12 years old	Most weight-loss supplements cannot be recommended at this time for children
10) Woo study/2009 [23]	Review	Review of pharmacological treatments for obesity, surgical options and recommendations for pediatric primary care	Lifestyle modification is the mainstay of obesity treatment for all ages. Pharmacotherapy reserved for specific adolescents	Therapies that are available to treat severely obese adolescents are discussed
11) Viner study/2009 [24]	Review	Review of population-based prescribing data from UK between 1999-2006	The annual prevalence of anti obesity drug prescriptions rose significantly in 2006, a 15-fold increase	Further research into the effectiveness and safety of antiobesity drugs in clinical populations of children and adolescents is needed
12) Elfhag study/2008 [18]	Retrospective study	Data was collected on 478 obese patients on different types of previous weight loss treatments	Sibutramine and orlistat both produced weight loss	Sibutramine exhibits its greatest effects in whom eating is a natural response to hunger
13) Uli study/2008 [22]	Review	Guidelines recommended by an expert panel were used	Staged-approach for treatment of childhood obesity is suggested	Health policy interventions can prevent obesity
14) Whitlock study/2008 [25]	Review	Two systematic reviews used	Medications + behavior change	Policy development is the key
15) Moya study/2008 [20]	Review	The methodology involved identification of pediatric population at risk	Treatment guides were presented along with other individual prevention studies	Involving motivated pediatricians and better follow-up in the frame of general national preventive program can be a rational outcome
16) McGovern study/2008 [28]	Systematic review	Data sources used were nine electronic databases from inception until Feb 2006	Short-term medications were effective, including sibutramine	There is a limited evidence of short-term efficacy of medications and lifestyle interventions
17) Baumer study/2007 [5]	Review	Public health guidance clinical guidelines produced	Two quick reference guides summarize the public health and clinical recommendations for both adults and children	Need for future research related to obesity prevention
18) Danielsson study/2007 [12]	A double-blind placebo controlled cross-over study	BMI scores were analyzed using a repeated-measures design (ANOVA)	Sibutramine treated group showed a significant decrease in BMI scores compared to placebo group (p < 0.001)	Sibutramine lead to clinically and statistically significant weight reduction in short-term.
19) Singhal study/2007 [7]	Review	Evaluation of dietary, pharmacological and surgical management of obesity	Lifestyle interventions and behavioral modification are essential for obesity management	Further evaluation research is needed.
20) Spear study/2007 [8]	Review	Various behaviors reviewed	4-staged weight approach suggested	Office-based behaviors need to be modified

## Role of Sibutramine

Sibutramine which was approved in 1998 has been widely evaluated in various trials since its approval. Among various placebo-controlled trials ranging from 16 - 52 weeks weight loss varied from 3.4 to 6 kilograms [9]. A large multicentered randomized trial from Sibutramine adolescent study group proved the drugs efficacy at par with previous studies including adults in decreasing adolescent BMI. Efficacy of sibutramine is also proven when it came to decrease in body weight, binge eating episodes and psychiatric symptoms. So there is emerging evidence that sibutramine can be a valid therapeutic option for obese adolescents. The only caveat being regular monitoring of blood pressure and pulse rates, though preliminary trials are reporting its safety and tolerability [10, 11]. In a double-blind placebo-controlled cross-over study conducted among children diagnosed with hypothalamic obesity, sibutramine was found to be efficacious [12]. This study may provide window of opportunities for using sibutramine in syndromic obesity in future.

Various studies discussing the role of sibutramine along with behavior therapy have shown successful results in obesity reduction [11]. As safety issues related to sibutramine therapy are still under investigation, trials conducted using intermittent therapies versus continuous therapy have a special role in obesity management. Intermittent therapy has shown similar efficacy to continuous drug administration [13].

## Role of Orlistat

The role of orlistat in adolescent overweight management is limited by the Food and Drug Administration for adolescents aged 12-18 years old and having BMI more than 2 units above the 95th percentile for the age and gender. A one year placebo-controlled trial where orlistat was used along with a hypo-caloric diet, exercise and behavior therapy showed a significant decrease in BMI along obese American adolescents [6]. The use of orlistat in pediatric patients is limited due to the severe side-effects such as fatty or oily stools along with a risk of possible malabsorption of fat soluble vitamins [6, 14]. Binge eating, a psychiatric condition leading to obesity has also been managed by using orlistat [15].

## Combined Trials: Sibutramine and Orlistat

The pharmacologic literature on the combination therapy of sibutramine with orlistat has shown great promise. In the first trial to assess efficacy of this combination, the patients were only randomized to added orlistat or placebo after a previous weight loss period with sibutramine alone. Addition of orlistat did not help in further weight loss that what was achieved with sibutramine alone [10]. Similarly, another randomized open-labeled short term trial failed to show any significant difference between the sibutramine only group and the combination (sibutramine + orlistat) group on decrease in BMI [16].

In a recent meta-analysis which included a total of eight trials (5 sibutramine and 3 orlistat), it was seen that there was a mean decrease in weight of 5.25 kilograms after a minimum follow-up period of 6 months [17]. In a study which tried to address whether there are any psychological correlates of combined drug therapy, it was concluded that sibutramine exerted its greatest effect in patients whose eating is a natural response to hunger than regulated by cognitions and conscious controls. Personality attribute of conscientiousness was important as orlistat needs good pharmacological adherence [18]. Thus we see that a combined pharmacological approach is affected by many extraneous factors such as personality and psychological characteristics of the persons involved.

Most of the literature which looks at a combined effect of sibutramine and olistat has found no additional benefit of adding orlistat in causing weight reduction in overweight adolescents. In a Cochrane review of 64 randomized-controlled trials, 10 studies focused on pharmacologic management of childhood obesity. The results concluded that pertaining to childhood obesity either sibutramine or orlistat can be used as an adjunct to life-style interventions. Side-effects of these drugs however should be carefully monitored [19]. Similarly a meta-analysis of 17 pharmacological interventions showed a large pooled effect size with a consistent loss in body mass index of 2.4 kg/m^2^ of sibutramine use in adolescent obesity reduction after 6 months of usage. While for orlistat, the reduction in BMI was 0.7 kg/m^2^, a small to moderate effect. This meta-analytic study reaffirms the earlier studies [28] in explicating short-term efficacy of both these drugs. The study of long term efficacy of these drugs continues to be a challenge among researchers, as the side-effect profile of these drugs precludes these designs.

## Discussion

Obesity and overweight management, particularly among adolescents and children continues to be a great challenge. The current management arsenal in the fight against obesity consists of dietary therapy, pharmacologic therapy and surgical intervention. The current review, focusing on two drugs sibutramine and orlistat, offers a panoramic view of the various trials and studies conducted till date. Though orlistat is approved to be used in adolescents, its use in pediatric population is associated with lot of problems such as fat malabsorption since it interferes with child growth. Similarly in adolescents the side-effect profile of this drug needs to be studied in details in long-term trials [20]. The results of the meta-analytic studies and placebo-controlled trials conducted till date give credence to the limited use of these drugs in adolescent obesity reduction. Studies which have shown usefulness of these drugs have either been short-term studies or have tried these pharmacological agents as an add-on treatment. The important questions to ask ourselves, as researchers, are what are the options we are having for childhood and adolescent obesity management? Dietary modifications and behavior therapies need a lot of parental input in terms of time and effort and weight-loss supplements make dubious claims of efficacy as often advertised [21]. Surgical intervention, bariatric surgery, is restricted to morbidly obese adolescents and is not a blanket option [22, 23]. This leaves us little choice in choosing an appropriate management strategy which is sustainable over time.

In conclusion, childhood obesity related pharmacological treatment has greatly increased in the past 8 years. The majority of these drugs including sibutramine and orlistat are rapidly discontinued before patients (children) can see the weight benefits [24]. This, itself is an evidence that drugs which have been having limited efficacy in adult population need to be used with greater scientific rigor and supervision in adolescent efficacy trials. Hence, in the light of the available evidence, it would be advisable and recommended to use these two drugs (sibutramine and orlistat) with caution and careful monitoring of side-effects. Current existing systematic reviews [25, 26] conclude a very limited short-term efficacy of these drugs and that too when they are added to the existing behavioral interventions. The need for long term efficacy trials and replication of current study protocols in future, cannot be overemphasized.

## Implications for Practice

It is very important to understand that adolescent obesity or overweight may be linked to multiple causes or having various associations such as hypothalamic obesity, binge eating disorders, anorexia nervosa and bulimia. The outcome of lifestyle interventions such as dietary modifications, increased physical activity and weight-loss supplements in obesity reduction is insufficient in present times. Pharmacological therapy holds promise for future in the form of drugs like sibutramine, orlistat and metformin. From the current practical standpoint, orlistat is the only FDA approved drug to be used in adolescents and its long-term effects are still questionable. Sibutramine, though used judiciously as an adult drug still needs to be accepted as a pediatric and an adolescent treatment drug due to its severe side-effects. Also long-term efficacy of this drug in consistent weight reduction needs to be studied in various adolescent populations with different dosages [27]. Future pharmacological studies involving sibutramine and orlistat will hold a definitive promise if researchers devise an altered molecular structure drug producing better tolerability and longer effect sustainability.

## References

[1] Ogden CL, Carroll MD, Flegal KM (2008). High body mass index for age among US children and adolescents, 2003-2006. JAMA.

[2] Kosti RI, Panagiotakos DB (2006). The epidemic of obesity in children and adolescents in the world. Cent Eur J Public Health.

[3] Kanekar A, Sharma M (2008). Meta-analysis of school-based childhood obesity interventions in the U.K. and U.S.. Int Q Community Health Educ.

[4] Olds TS (2009). One million skinfolds: secular trends in the fatness of young people 1951-2004. Eur J Clin Nutr.

[5] Baumer JH (2007). Obesity and overweight: its prevention, identification, assessment and management. Arch Dis Child Educ Pract Ed.

[6] Godoy-Matos AF, Guedes EP, Souza LL, Martins MF (2009). Management of obesity in adolescents: state of art. Arq Bras Endocrinol Metabol.

[7] Singhal V, Schwenk WF, Kumar S (2007). Evaluation and management of childhood and adolescent obesity. Mayo Clin Proc.

[8] Spear BA, Barlow SE, Ervin C, Ludwig DS, Saelens BE, Schetzina KE, Taveras EM (2007). Recommendations for treatment of child and adolescent overweight and obesity. Pediatrics.

[9] Li Z, Maglione M, Tu W, Mojica W, Arterburn D, Shugarman LR, Hilton L (2005). Meta-analysis: pharmacologic treatment of obesity. Ann Intern Med.

[10] Coutinho W (2009). The first decade of sibutramine and orlistat: a reappraisal of their expanding roles in the treatment of obesity and associated conditions. Arq Bras Endocrinol Metabol.

[11] Tziomalos K, Krassas GE, Tzotzas T (2009). The use of sibutramine in the management of obesity and related disorders: an update. Vasc Health Risk Manag.

[12] Danielsson P, Janson A, Norgren S, Marcus C (2007). Impact sibutramine therapy in children with hypothalamic obesity or obesity with aggravating syndromes. J Clin Endocrinol Metab.

[13] Wirth A, Krause J (2001). Long-term weight loss with sibutramine: a randomized controlled trial. JAMA.

[14] Maahs D, de Serna DG, Kolotkin RL, Ralston S, Sandate J, Qualls C, Schade DS (2006). Randomized, double-blind, placebo-controlled trial of orlistat for weight loss in adolescents. Endocr Pract.

[15] Golay A, Laurent-Jaccard A, Habicht F, Gachoud JP, Chabloz M, Kammer A, Schutz Y (2005). Effect of orlistat in obese patients with binge eating disorder. Obes Res.

[16] Kaya A, Aydin N, Topsever P, Filiz M, Ozturk A, Dagar A, Kilinc E (2004). Efficacy of sibutramine, orlistat and combination therapy on short-term weight management in obese patients. Biomed Pharmacother.

[17] Czernichow S, Lee CM, Barzi F, Greenfield JR, Baur LA, Chalmers J, Woodward M (2009). Efficacy of weight loss drugs on obesity and cardiovascular risk factors in obese adolescents: a meta-analysis of randomized controlled trials. Obes Rev.

[18] Elfhag K, Finer N, Rossner S (2008). Who will lose weight on sibutramine and orlistat? Psychological correlates for treatment success. Diabetes Obes Metab.

[19] Meleti M, Leemans CR, Mooi WJ, Vescovi P, van der Waal I (2007). Oral malignant melanoma: a review of the literature. Oral Oncol.

[20] Moya M (2008). An update in prevention and treatment of pediatric obesity. World J Pediatr.

[21] Rogovik AL, Goldman RD (2009). Should weight-loss supplements be used for pediatric obesity?. Can Fam Physician.

[22] Uli N, Sundararajan S, Cuttler L (2008). Treatment of childhood obesity. Curr Opin Endocrinol Diabetes Obes.

[23] Woo T (2009). Pharmacotherapy and surgery treatment for the severely obese adolescent. J Pediatr Health Care.

[24] Viner RM, Hsia Y, Neubert A, Wong IC (2009). Rise in antiobesity drug prescribing for children and adolescents in the UK: a population-based study. Br J Clin Pharmacol.

[25] Gonzalez-Garcia R, Naval-Gias L, Martos PL, Nam-Cha SH, Rodriguez-Campo FJ, Munoz-Guerra MF, Sastre-Perez J (2005). Melanoma of the oral mucosa. Clinical cases and review of the literature. Med Oral Patol Oral Cir Bucal.

[26] Whitlock EP, O'Connor EA, Williams SB, Beil TL, Lutz KW (2010). Effectiveness of weight management interventions in children: a targeted systematic review for the USPSTF. Pediatrics.

[27] Rogovik AL, Chanoine JP, Goldman RD (2010). Pharmacotherapy and weight-loss supplements for treatment of paediatric obesity. Drugs.

[28] McGovern L, Johnson JN, Paulo R, Hettinger A, Singhal V, Kamath C, Erwin PJ (2008). Clinical review: treatment of pediatric obesity: a systematic review and meta-analysis of randomized trials. J Clin Endocrinol Metab.

